# Chemistry as a Servant of Anatomy

**Published:** 1895-10

**Authors:** 


### Chemistry as the Servant of Anatomy.

The French Institute has just elected as a foreign associate Professor Kowalevsky, of St. Petersburg, whose original and novel experiments in anatomy and biology are known to all students of these sciences. Among other ingenious methods of experimentation he has devised what may be called that of "chemical anatomy;" that is, a system of recognizing certain organs in the lower animals by observing the reactions in them of appropriate injected chemical substances. By this method he has arrived at results that could never have been attained by dissection alone, even with the aid of the most powful microscope. We translate from a notice of Kowalevsky's work in the *Revue Scientifique*, Paris, August 3, an account of some of these experiments:

"M. Kowalevsky has established the most curious and unexpected distinctions; thus by means of experiments of great elegance and simplicity he has succeeded in recognizing in invertebrates the kidneys, the lymphatic glands, and the spleen, though the scalpel of ordinary anatomy would have been powerless to discover them. His method is very simple. He injects into the body of the animal colored liquids like carminate of ammonia, indigo carmine, the classic dye of heliotrope, chlorid of iron, or impalpable powders such as the carmine or black suspended in India ink, and sometimes the bacteria of charbon, which he cultivates. He lets the animal live for a longer or shorter time and then kills it and shows what has happened to the injected material.

"One or two examples, taken from innumerable experiments, will suffice to show the precision of the method.

"The tincture of heliotrope injected into a cuttlefish remains blue in the majority of the organs of the body, notably in the multiple appendices situated in front of the branchial hearts; but in these last organs it changes to red; a little ammonia, even its vapor alone, changes it back to blue.

"These branchial hearts have, then, another function than the purely mechanical one; they secrete an acid.

"The choice and picking out of the reagents by the organism is yet much more remarkable in the following experiment.

"Into a St. James's snail was injected an intimate mixture of carminate of ammonia and indigo carmine. The animal was allowed to live for some time and then dissected.

It is well known that in this mollusk there are glands near the heart, called the precardial glands, and two other much larger glands placed on each side of the visceral mass, called the bodies of Bojanus.

"What action has each of these glands on these reagents? The carminate of ammonia remains in the precardial glands, which give an acid reaction after the injection of heliotrope; the indigo carmine is found in the body of Bojanus.

"The precardial glands are, then, the homologues of the cortical layer of the kidneys, where are found the Malpighian bodies, having an acid reaction, while the bodies of Bojanus, with their alkaline reaction, . . . correspond to the zone of the *tubuli contorti.*

"It is useless to pursue further the analysis of this method of chemical anatomy. Nevertheless it is impossible not to recall how happily injections of chlorid of iron serve in the diagnosis of some organs. For in exploring the organism of the animal that has been given an injection, we can, by the acid of yellow prusiate of potash and of the blue color that it gives with iron, recognize unmistakably where the iron has collected and where it has left no trace. . . .

"The consequence of these studies is the discovery of the duplication and division of several glands, and of organs that anatomy and the scalpel alone had not revealed to us and could not possibly have discovered. M. Kowalevsky has thus revealed to us scattered groups of cells, or even isolated cells, that represent the most complete organs.

"Thus, by this method he has just sought for the lymphatic glands of myriapods and has found them scattered about in the form of groups of cells or of isolated cells on the sides of the body or elsewhere, where they had never hitherto been recognized."—*Translated for* THE LITERARY DIGEST.

